# Supplementary material for: TRPV1 antagonism occurs through diverse structural mechanisms
Source: bioRxiv. 2026 Apr 29:2026.04.27.721197. Preprint. [Version 1] doi: 10.64898/2026.04.27.721197 (PMC13142412; doi:10.64898/2026.04.27.721197)
Supplement: Supplement 1 [file media-1.docx]

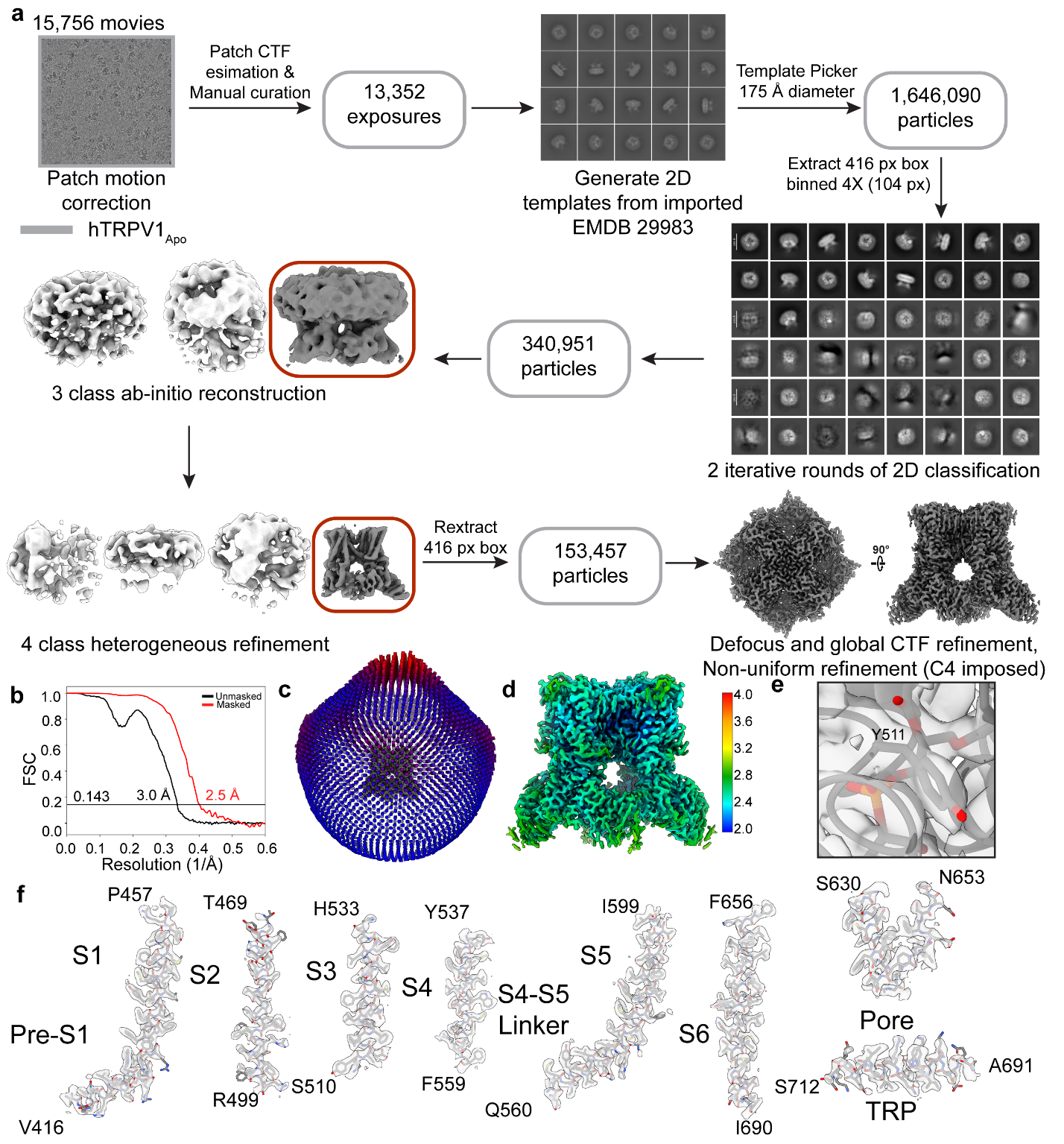


**Supplementary Figure 1: hTRPV1_Apo_ cryo-EM data processing collection and validation. a** Data collection and processing workflow for hTRPV1_Apo_. **b** Fourier shell correlation for the final map refinement with masked (red) and unmasked (black) curves displayed and the resolution using the gold standard cutoff (0.143) marked with a horizontal black line. **c** Euler angle distribution plot. **d** Local resolution estimates. **e** Cryo-EM density for Y511 where the residue and PI lipid are shown as sticks. **f** Map and model for each transmembrane helix with every residue represented as sticks.


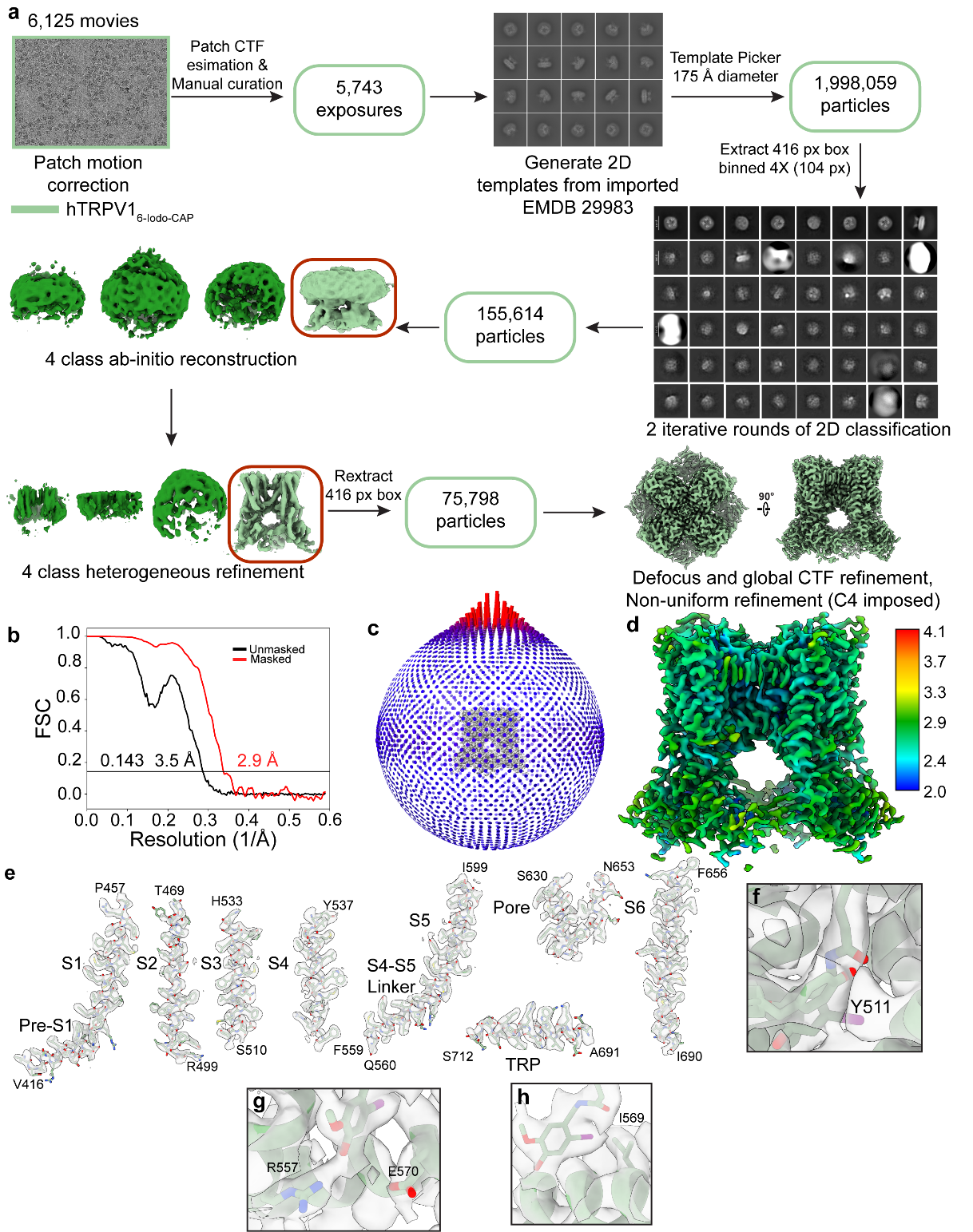


**Supplementary Figure 2: hTRPV1_6-Iodo-CAP_ cryo-EM data collection, processing, and validation. a** Data collection and processing workflow for hTRPV1_6-Iodo-CAP_. **b** Fourier shell correlation for the final map refinement with masked (red) and unmasked (black) curves displayed and the resolution using the gold standard cutoff (0.143) marked with a horizontal black line. **c** Euler angle distribution plot. **d** Local resolution estimate. **e** Map and model for each transmembrane helix with every residue represented as sticks. Density for residues Y511 (**f**), R557 and E570 (**g**), and I569 (**i**) with residues labeled and represented as sticks.


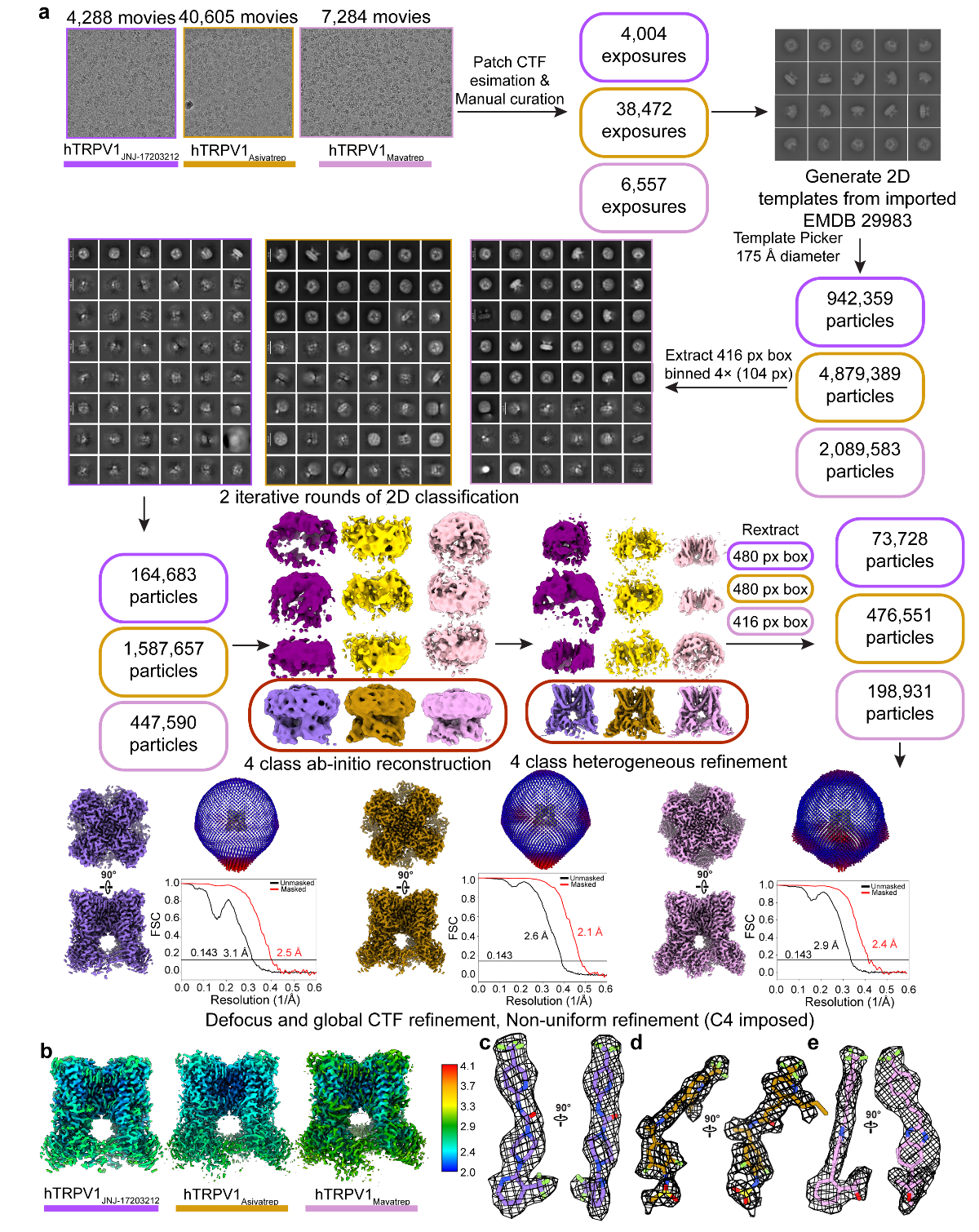


**Supplementary Figure 3: hTRPV1_JNJ-17203212_, hTRPV1_Asivatrep_, hTRPV1_Mavatrep_ cryo-EM data collection, processing and validation. a** Data collection and processing workflow for hTRPV1_JNJ-17203212_ (purple), hTRPV1_Asivatrep_ (orange, and hTRPV1_Mavatrep_ (pink). Cryo-EM map top and bottom views displayed in corresponding colors, Euler angle distribution plots and FSC plots for masked (red) and unmasked (black) displayed and gold standard resolution cutoff (0.143) shown. **b** Local resolution estimates for hTRPV1_JNJ-17203212_, hTRPV1_Asivatrep_, and hTRPV1_Mavatrep_ with labels for each structure underneath. Ligand model and density for JNJ-17203212 (**c**), Asivatrep (**d**), and Mavatrep (**e**).


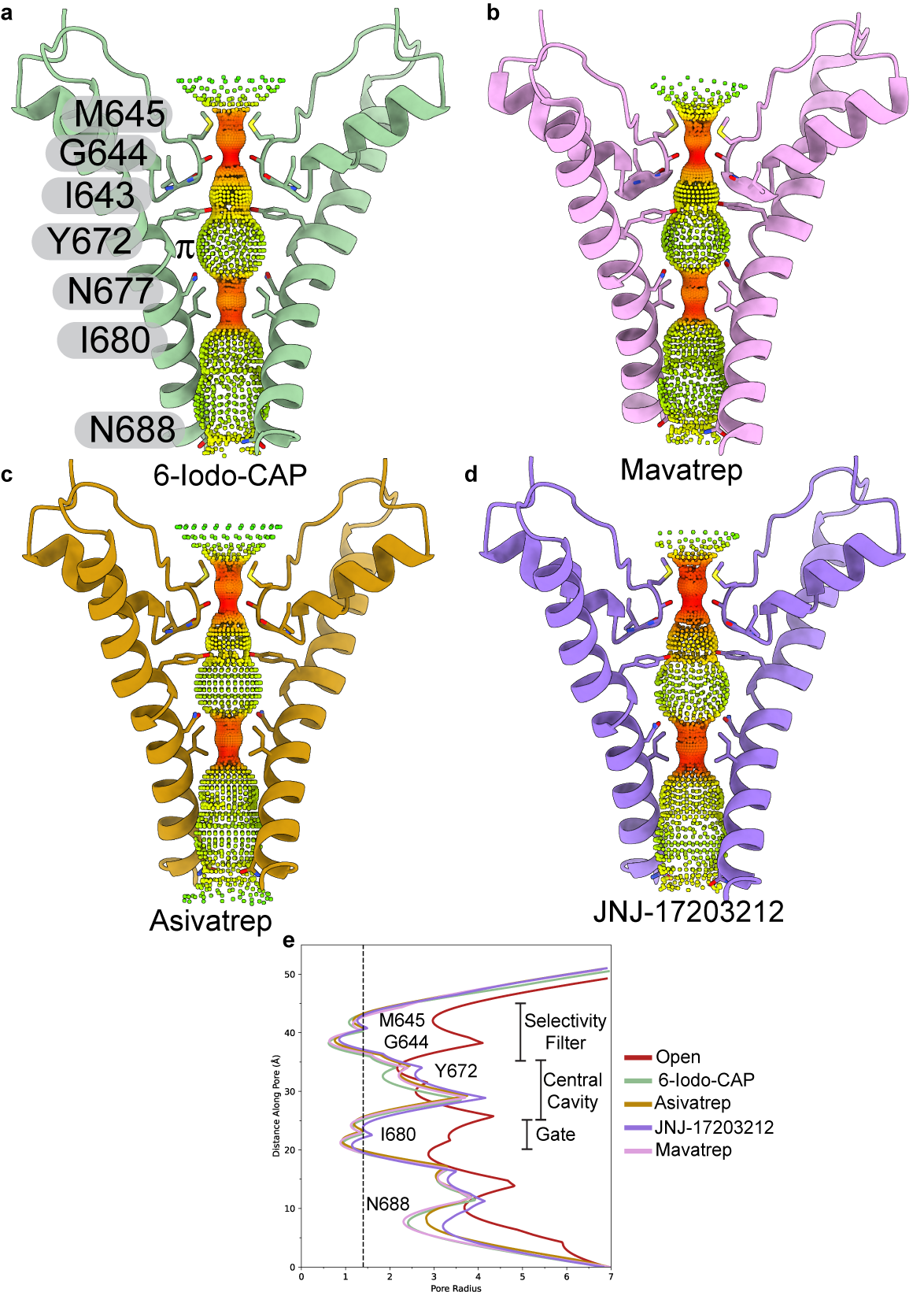


**Supplementary Figure 4: Pore analysis for cryo-EM structures.** HOLE analysis where radius size colored red (widest), yellow (intermediate) and green (narrowest) and pore lining residues are displayed and labeled for hTRPV1_6-Iodo-CAP_ (**a**), hTRPV1_Mavatrep_ (**b**), hTRPV1_Asivatrep_ (**c**), and hTRPV1_JNJ-17203212_ (**d**). **e** Plot for HOLE analysis for our structures and the rat TRPV1 open state structure (red; PDB 5IRX). Pore lining residues are labeled, and the radius of a water molecule is represented by a dashed line (1.4 Å).

**
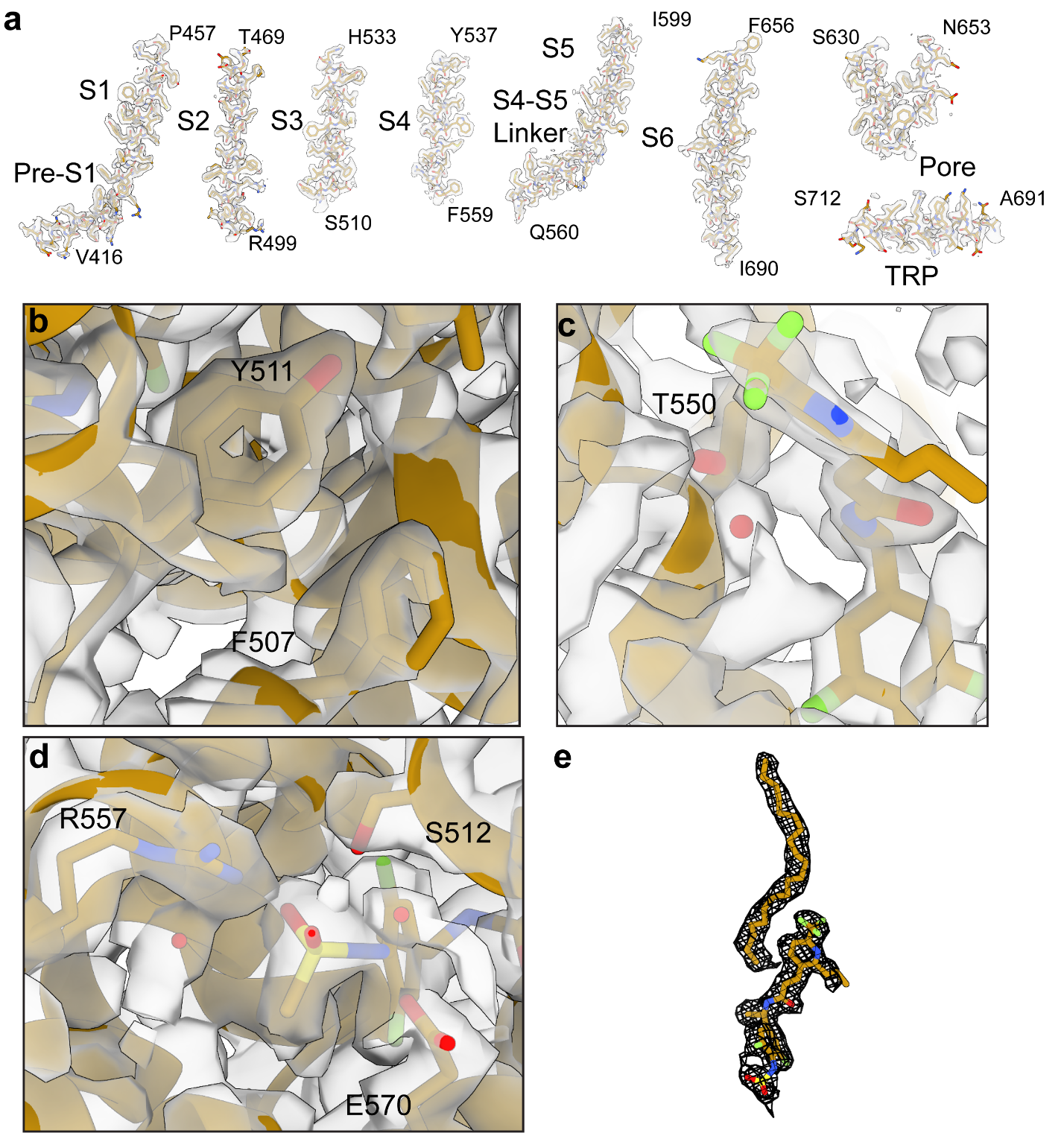
**

**Supplementary Figure 5: Cryo-EM structural analysis of hTRPV1_Asivatrep_. a** Map and model for each transmembrane helix with every residue represented as sticks. Cryo-EM densities for Y511 and F507 (**b**), T550 and bound water molecule (**c**), and R557, S512, E570 and bound water molecules (**d**). **e** Cryo-EM density (black mesh) and model (orange) for Asivatrep ligand (bottom) and eicosane molecule (top).


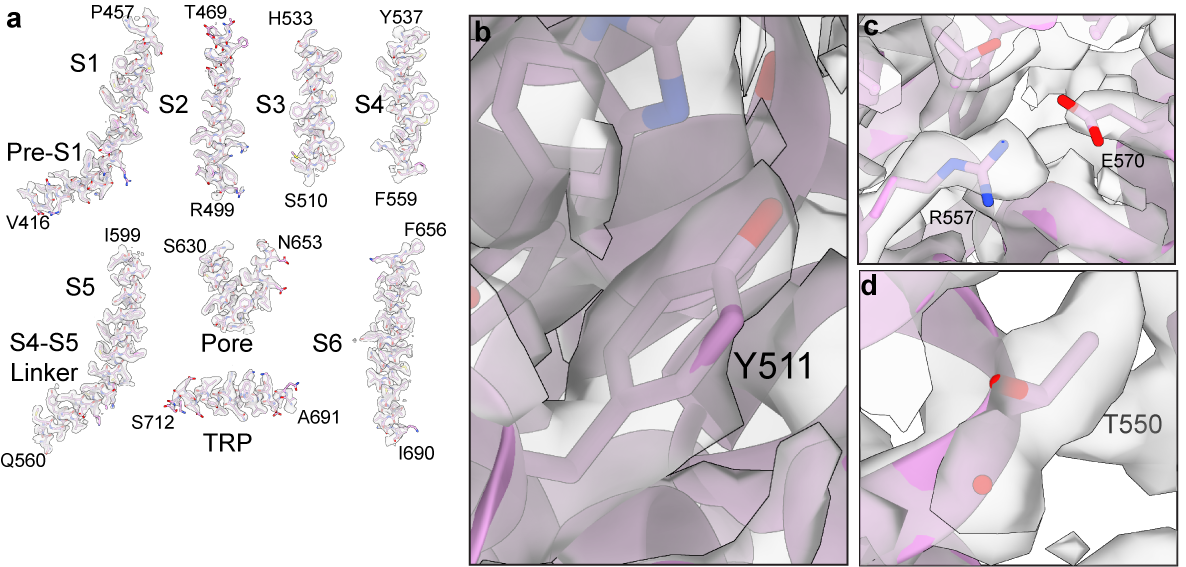


**Supplementary Figure 6: Cryo-EM structural analysis of hTRPV1_Mavatrep_. a** Map and model for each transmembrane helix with every residue represented as sticks. Cryo-EM density and model shown for Y511 (**b**), R557 and E570 (**c**), and T550 and a water molecule (**d**).


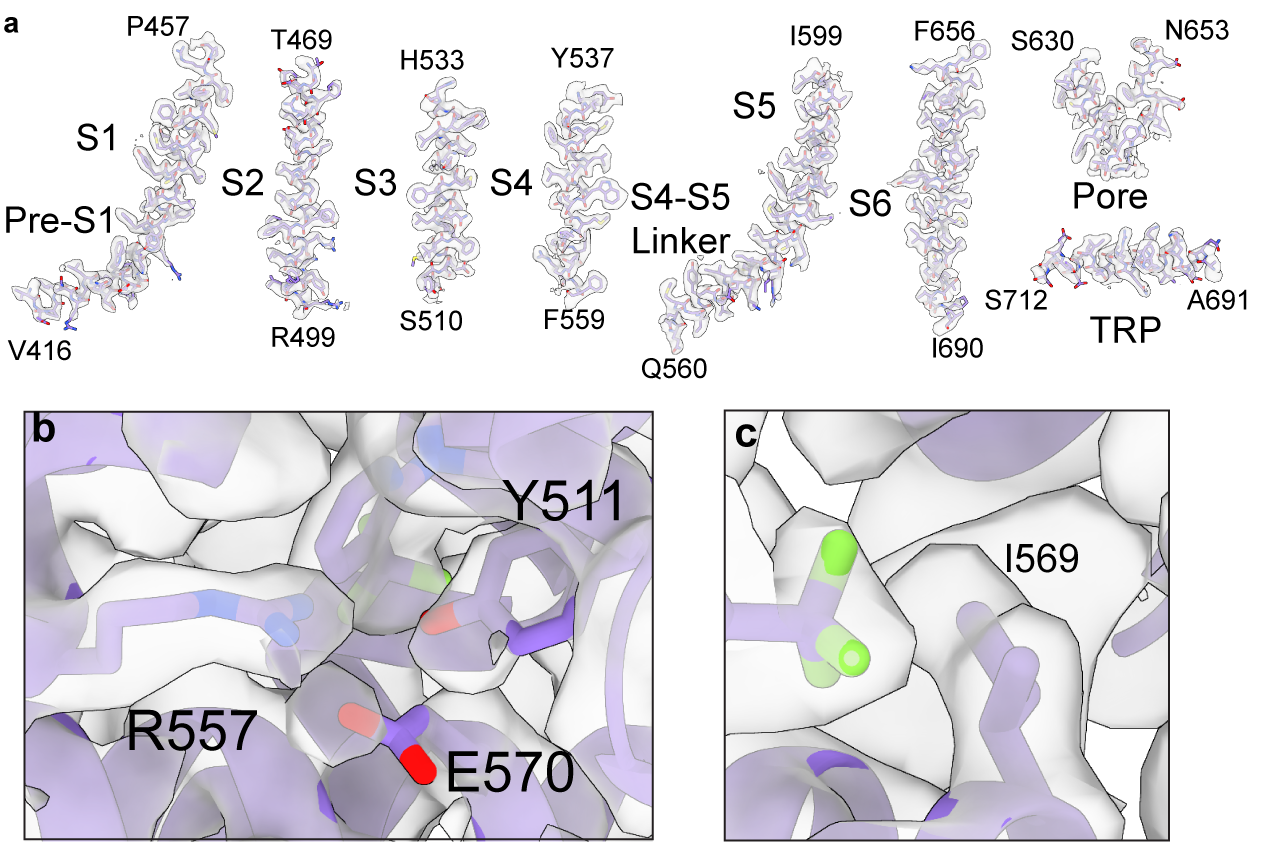


**Supplementary Figure 7: Cryo-EM structural analysis of hTRPV1_JNJ-17203212_. a** Map and model for each transmembrane helix with every residue represented as sticks. Cryo-EM density and model shown for R557, E570, and Y511 (**b**) and I569 (**c**).

**
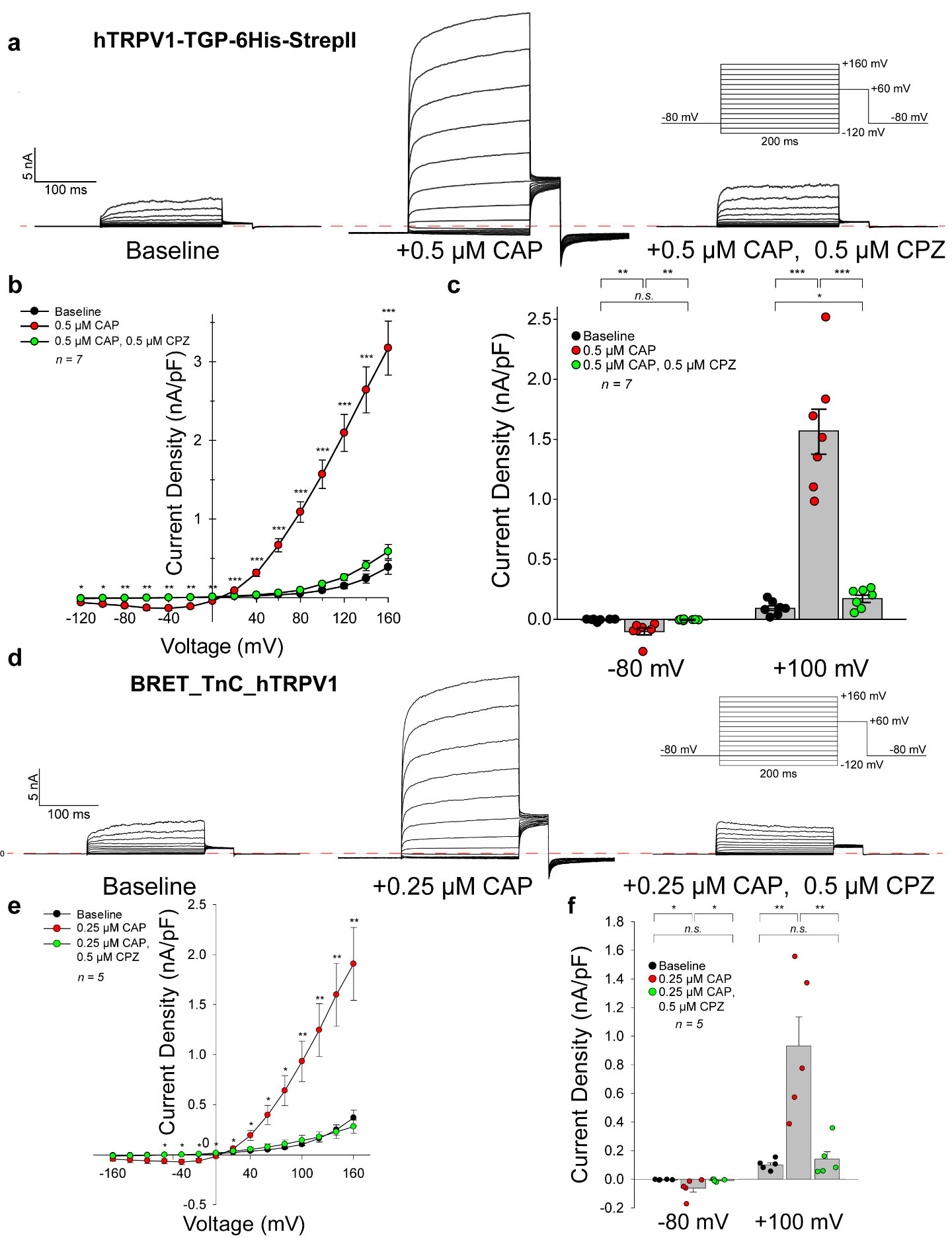
**

**Supplementary Figure 8: Functional characterization of TRPV1 constructs. a** Averaged (n = 7) whole-cell manual patch-clamp recordings for structural construct hTRPV1-TGP-6His-StrepII using a voltage-clamp step protocol from -120 mV to +160 mV in 20 mV increments. Sweeps show baseline current, current response during perfusion of 500 nM capsaicin (CAP), and current response during subsequent co-application of 500 nM CAP and 500 nM capsazepine (CPZ). **b** Current-voltage (IV) plot generated from the sweeps shows averaged current density at the 300 ms time point for each of the voltage steps under baseline conditions (black), CAP activation (red), and CAP + CPZ co-application (green). Error is the standard error of the mean. Statistical significance was determined using a student’s t-test to compare the CAP and CAP + CPZ current response. P ≤ 0.05 (*), P ≤ 0.01 (**), P ≤ 0.001 (***). **c** Bar plots show the averaged current density at the -80 mV and +100 mV voltage steps with individual replicates jittered to show the spread of cell current responses. Error is the standard error of the mean. **d** Averaged (n = 5) whole-cell manual patch-clamp recordings for BRET construct BRET_TnC_hTRPV1 using a voltage-clamp strep protocol from -120 mV to +160 mV in 20 mV increments. Sweeps show baseline current, current response during perfusion of 250 nM CAP and 500 nM CPZ. **e** Current-voltage (IV) plot generated from the sweeps shows averaged current density at the 300 ms time point for each of the voltage steps under baseline conditions (black), CAP activation (red), and CAP + CPZ co-application (green). Error is the standard error of the mean. Statistical significance was determined in the same way as (**b**). **f** Bar plots show the averaged current density at the -60 mV and +100 mV voltage steps with individual replicates jittered to show the spread of cell current response. Error is the standard error of the mean.


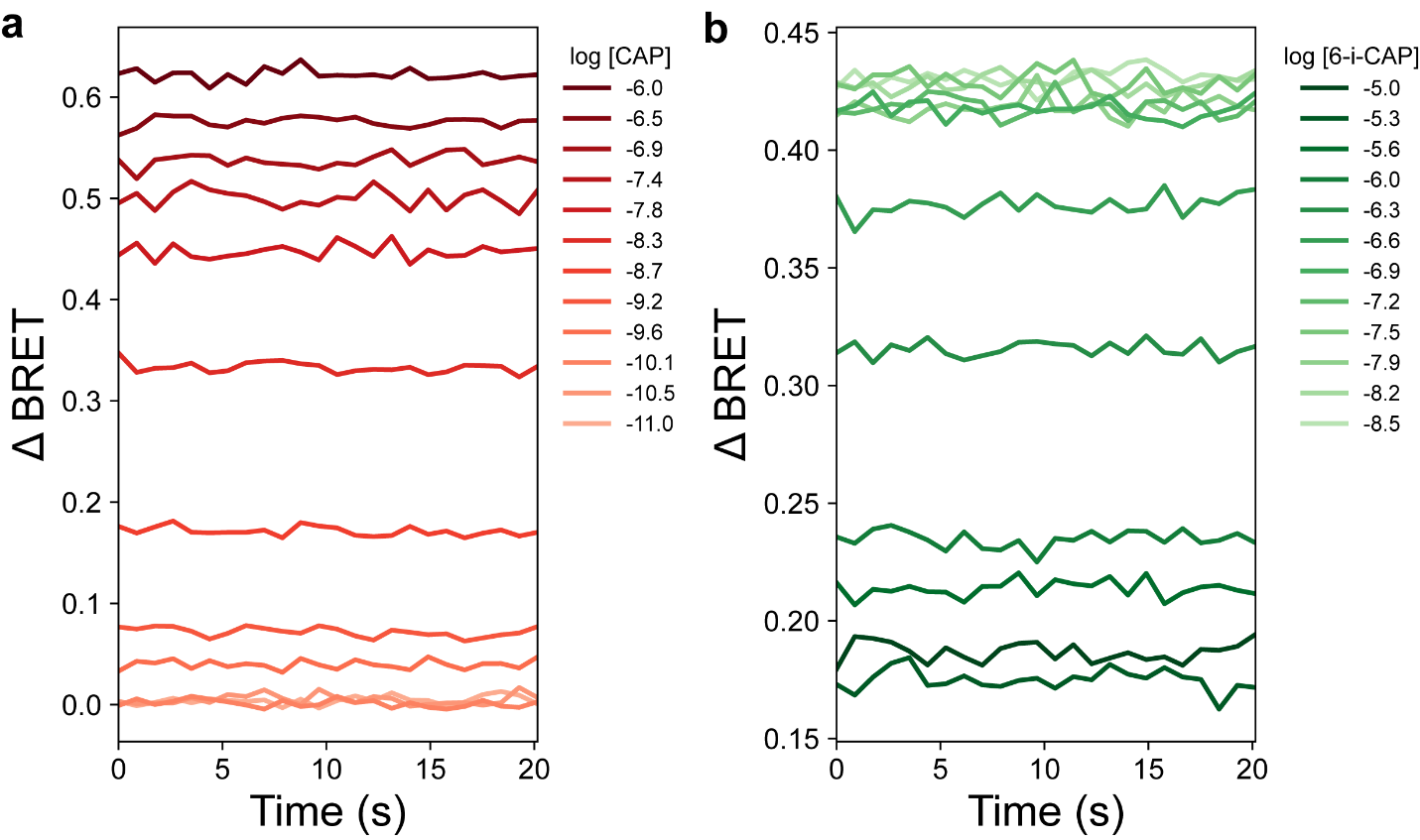


**Supplementary Figure 9: BRET time course for Capsaicin and 6-Iodo-CAP.** ΔBRET signal over a 20 s time course with increasing concentrations of CAP (**a**) and 6-Iodo-CAP (**b**). Traces represent the average of 12 replicates per concentration for both CAP and 6-Iodo-CAP.

**Supplementary Table 1 Cryo-EM data collection, refinement and validation statistics**

|  | hTRPV1_Apo_  (EMDB-75616)  (PDB 11CJ) | hTRPV1_6-Iodo-CAP_  (EMDB-75617)  (PDB 11CK) | hTRPV1_Mavatrep_  (EMDB-75619)  (PDB 11CN) |
| --- | --- | --- | --- |
| **Data collection and processing** |  |  |  |
| Magnification | 165,000 | 105,000 | 105,00 |
| Voltage (kV) | 300 | 300 | 300 |
| Electron exposure (e–/Å^2^) | 50 | 50 | 50 |
| Defocus range (μm) | 1.5-2.5 | 1.5-2.5 | 0.8-2.5 |
| Pixel size (Å) | 0.73 | 0.83 | 0.83 |
| Symmetry imposed | C4 | C4 | C4 |
| Initial particle images (no.) | 1,646,090 | 1,660,569 | 2,089,583 |
| Final particle images (no.) | 153,457 | 75,798 | 198,931 |
| Map resolution (Å)  FSC threshold | 2.52  0.143 | 2.90  0.143 | 2.37  0.143 |
| Map resolution range (Å) | 2.0-2.9 | 2.2-3.2 | 2.2-3.1 |
|  |  |  |  |
| **Refinement** |  |  |  |
| Initial model used (PDB code) | 8GF8 | 11CN | 8GFA |
| Model resolution (Å)  FSC threshold | 2.51  0.143 | 2.90  0.143 | 2.36  0.143 |
| Map sharpening *B* factor (Å^2^) | -75.5 | -83.6 | -78.6 |
| Model composition  Non-hydrogen atoms  Protein residues  Ligands | 17,684  2,124  8 | 17,340  2,124  4 | 17,376  2,124  4 |
| *B* factors (min/max/mean Å^2^)  Protein  Ligand  Water | 1.00/120.37/35.49  12.62/108.43/47.15  N/A | 12.79/152.08/78.75  25.78/125.88/44.29  N/A | 5.39/144.74/56.58  9.41/41.73/20.08  20.7/20.7/20.7 |
|  |  |  |  |
| R.m.s. deviations  Bond lengths (Å)  Bond angles (°) | 0.004  0.912 | 0.004  0.904 | 0.004  0.959 |
| Validation  MolProbity score  Clashscore  Poor rotamers (%) | 1.56  5.17  0.7 | 1.47  5.50  0.54 | 1.71  9.23  0.48 |
| Ramachandran plot  Favored (%)  Allowed (%)  Disallowed (%) | 95.83  4.17  0 | 97.01  3.12  0 | 96.58  3.12  0.19 |

**Supplementary Table 1 (continued)**

|  | hTRPV1_Asivatrep_  (EMDB-75618)  (PDB 11CL) | hTRPV1_JNJ-17203212_  (EMDB-75620)  (PDB 11CO) |
| --- | --- | --- |
| **Data collection and processing** |  |  |
| Magnification | 165,00 | 165,000 |
| Voltage (kV) | 300 | 300 |
| Electron exposure (e–/Å^2^) | 50 | 20 |
| Defocus range (μm) | 1.5-2.5 | 0.5-1.5 |
| Pixel size (Å) | 0.73 | 0.76 |
| Symmetry imposed | C4 | C4 |
| Initial particle images (no.) | 4,879,389 | 942,359 |
| Final particle images (no.) | 476,551 | 73,728 |
| Map resolution (Å)  FSC threshold | 2.10  0.143 | 2.49  0.143 |
| Map resolution range (Å) | 2.0-3.0 | 2.4-3.5 |
|  |  |  |
| **Refinement** |  |  |
| Initial model used (PDB code) | 11CN | 11CN |
| Model resolution (Å)  FSC threshold | 2.08  0.143 | 2.47  0.143 |
| Map sharpening *B* factor (Å^2^) | -65.3 | -67.1 |
| Model composition  Non-hydrogen atoms  Protein residues  Ligands | 17,476  2,124  8 | 17,364  2,124  4 |
| *B* factors (min/max/mean Å^2^)  Protein  Ligand | 0.14/117.06/43.70  6.03/46.38/22.26 | 2.13/162.89/62.93  15.07/45.91/29.29 |
| Water | 17.22/29.57/21.54 | N/A |
| R.m.s. deviations  Bond lengths (Å)  Bond angles (°) | 0.004  0.866 | 0.004  0.91 |
| Validation  MolProbity score  Clashscore  Poor rotamers (%) | 1.22  2.86  0.21 | 1.34  2.91  0 |
| Ramachandran plot  Favored (%)  Allowed (%)  Disallowed (%) | 97.25  2.75  0 | 96.2  3.8  0 |
