# Supplementary material for: TRPV1 antagonism occurs through diverse structural mechanisms
Source: bioRxiv. 2026 Apr 29:2026.04.27.721197. Preprint. [Version 1] doi: 10.64898/2026.04.27.721197 (PMC13142412; doi:10.64898/2026.04.27.721197)
Supplement: Supplement 2 [file media-2.doc]

**Cryo-EM data collection, refinement and validation statistics**

|  | hTRPV1_Apo_  (EMDB-75616)  (PDB 11CJ) | hTRPV1_6-Iodo-CAP_  (EMDB-75617)  (PDB 11CK) | hTRPV1_Mavatrep_  (EMDB-75619)  (PDB 11CN) |
| --- | --- | --- | --- |
| **Data collection and processing** |  |  |  |
| Magnification | 165,000 | 105,000 | 105,00 |
| Voltage (kV) | 300 | 300 | 300 |
| Electron exposure (e–/Å^2^) | 50 | 50 | 50 |
| Defocus range (μm) | 1.5-2.5 | 1.5-2.5 | 0.8-2.5 |
| Pixel size (Å) | 0.73 | 0.83 | 0.83 |
| Symmetry imposed | C4 | C4 | C4 |
| Initial particle images (no.) | 1,646,090 | 1,660,569 | 2,089,583 |
| Final particle images (no.) | 153,457 | 75,798 | 198,931 |
| Map resolution (Å)  FSC threshold | 2.52  0.143 | 2.90  0.143 | 2.37  0.143 |
| Map resolution range (Å) | 2.0-2.9 | 2.2-3.2 | 2.2-3.1 |
|  |  |  |  |
| **Refinement** |  |  |  |
| Initial model used (PDB code) | 8GF8 | 11CN | 8GFA |
| Model resolution (Å)  FSC threshold | 2.51  0.143 | 2.90  0.143 | 2.36  0.143 |
| Map sharpening *B* factor (Å^2^) | -75.5 | -83.6 | -78.6 |
| Model composition  Non-hydrogen atoms  Protein residues  Ligands | 17,684  2,124  8 | 17,340  2,124  4 | 17,376  2,124  4 |
| *B* factors (min/max/mean Å^2^)  Protein  Ligand  Water | 1.00/120.37/35.49  12.62/108.43/47.15  N/A | 12.79/152.08/78.75  25.78/125.88/44.29  N/A | 5.39/144.74/56.58  9.41/41.73/20.08  20.7/20.7/20.7 |
|  |  |  |  |
| R.m.s. deviations  Bond lengths (Å)  Bond angles (°) | 0.004  0.912 | 0.004  0.904 | 0.004  0.959 |
| Validation  MolProbity score  Clashscore  Poor rotamers (%) | 1.56  5.17  0.7 | 1.47  5.50  0.54 | 1.71  9.23  0.48 |
| Ramachandran plot  Favored (%)  Allowed (%)  Disallowed (%) | 95.83  4.17  0 | 97.01  3.12  0 | 96.58  3.12  0.19 |

**Cryo-EM data collection, refinement and validation statistics**

|  | hTRPV1_Asivatrep_  (EMDB-75618)  (PDB 11CL) | hTRPV1_JNJ-17203212_  (EMDB-75620)  (PDB 11CO) |
| --- | --- | --- |
| **Data collection and processing** |  |  |
| Magnification | 165,00 | 165,000 |
| Voltage (kV) | 300 | 300 |
| Electron exposure (e–/Å^2^) | 50 | 20 |
| Defocus range (μm) | 1.5-2.5 | 0.5-1.5 |
| Pixel size (Å) | 0.73 | 0.76 |
| Symmetry imposed | C4 | C4 |
| Initial particle images (no.) | 4,879,389 | 942,359 |
| Final particle images (no.) | 476,551 | 73,728 |
| Map resolution (Å)  FSC threshold | 2.10  0.143 | 2.49  0.143 |
| Map resolution range (Å) | 2.0-3.0 | 2.4-3.5 |
|  |  |  |
| **Refinement** |  |  |
| Initial model used (PDB code) | 11CN | 11CN |
| Model resolution (Å)  FSC threshold | 2.08  0.143 | 2.47  0.143 |
| Map sharpening *B* factor (Å^2^) | -65.3 | -67.1 |
| Model composition  Non-hydrogen atoms  Protein residues  Ligands | 17,476  2,124  8 | 17,364  2,124  4 |
| *B* factors (min/max/mean Å^2^)  Protein  Ligand | 0.14/117.06/43.70  6.03/46.38/22.26 | 2.13/162.89/62.93  15.07/45.91/29.29 |
| Water | 17.22/29.57/21.54 | N/A |
| R.m.s. deviations  Bond lengths (Å)  Bond angles (°) | 0.004  0.866 | 0.004  0.91 |
| Validation  MolProbity score  Clashscore  Poor rotamers (%) | 1.22  2.86  0.21 | 1.34  2.91  0 |
| Ramachandran plot  Favored (%)  Allowed (%)  Disallowed (%) | 97.25  2.75  0 | 96.2  3.8  0 |
